# Supplementary material for: A Nickel-Containing Polyoxomolybdate as an Efficient Antibacterial Agent for Water Treatment
Source: Int J Mol Sci. 2022 Aug 25;23(17):9651. doi: 10.3390/ijms23179651 (PMC9456081; doi:10.3390/ijms23179651)
Supplement: Supplementary file 1 [file ijms-23-09651-s001.zip › ijms-1835452-supplementary.pdf]

A nickel-containing polyoxomolybdate as an efficient antibacterial agent for water treatment

Jiangnan Chang†, Mingxue Li†, Jiyuan Du, Min Ma, Cuili Xing, Lin Sun* and Pengtao Ma*

Henan Key Laboratory of Polyoxometalate Chemistry, School of Chemistry and Chemical Engineering, Henan University, Kaifeng, Henan, 475004, China

* Correspondence: sunlin@vip.henu.edu.cn (L.S.); mpt@henu.edu.cn (P.M.)

† These authors contributed equally to this work.

1. Materials and methods

1.1 X-ray crystallography

Single-crystal X-ray crystallography Suitable single crystals of **1** with dimensions of 0.30 × 0.21 × 0.16 mm^3, were picked on an optical microscope and sealed to glass tubes. Crystallographic data for **1** were collected on a Bruker SMART-CCD Apex-II diffractometer with Mo Kα radiation (λ = 0.71073 Å) at 296(2) K. Using Olex2 [28], the structure was solved with the ShelXT structure solution program using Intrinsic Phasing and refined with the ShelXL refinement package using Least Squares minimization on F^2 [29]. Anisotropic displacement parameters were refined for all non-hydrogen atoms. Hydrogen atoms on ligands are added in the riding model. CCDC-2119325 for **1** has been accessed from Cambridge Crystallographic Data. Crystal data and refinement parameters are given in Table S1.

1.2 Antibacterial activity test

In this study, disc diffusion method was implemented to assess the antibacterial activity of **1** against *E. coli*, *A. tumefaciens* and *S. aureus*, *B. subtilis*, and noticeable results were determined by the MIC. Firstly, all the materials used in the testing process were sterilized by ultraviolet radiation for 30 min. Subsequently, 0.1 mL of bacterial suspension of four strains (10^7 colony forming units (CFU)/mL) was spread equably on the superficies of the nutrient agar. Following the preparation of the original concentration of **1** (1 mg/mL), different concentrations were obtained by two-fold dilution in this assay. 1 mg/mL solution of **1** was prepared using dimethyl sulfoxide (DMSO) solvents. On the basis of 1 mg/mL solution, a series of solutions with different concentrations were obtained by a double step-by-step dilution method using DMSO solvents.

Subsequently, each separate well was filled with **1** of 100 μl and the plates were incubated at 37 °C for 24 h. While the bacteria without material treatment was selected as blank control group, antibacterial experiments were performed on a UV-ultra-net workbench. In addition, each antibacterial assay was repeated three times. Finally, the zones of the inhibition (ZOI) along with the optical images were recorded for all samples.

1.3 Time-kill studies

Through time-kill studies, the antibacterial response of **1** has been further characterized and the colony counting method has been used to determine the bacterial growth inhibition. To compare the results, the untreated bacterial strains were used as controls. Firstly, the same volume of bacteria (10^5 (CFU)/mL) and **1** were incubated in an incubator shaker for different times (2, 4, 6 h). From this times onwards, the treated bacterial suspension (100 μL) was equably coated on the nutrient agar plate and cultured in an incubator at 37 ℃ for 16 hours. Besides, the experiments were repeated three times and the survival rate of bacterial can be calculated by Equ 1, colony images were obtained using a digital camera. Where N is the colony number of the control group and N_1 is the colony number of the test group.

Equ S1 cells (%)=$N-N_1/ N \times 100\%$

1.4 Adsorption performance of 1

The adsorption capacity of **1** was established by the removal of organic dye in dark under room temperature. Briefly, certain amounts (20 mg) of **1** were added to 50 mL of BF solution (15 mg/L) and stirred continuously. After specific time intervals, 3 mL of the dye solution was taken out, centrifuged for 4 minutes at 3500 rpm, and the absorbance at 542 nm was measured with a UV-Vis spectrophotometer (TU-1900, China). The removal efficiency (%) were calculated by the Equ2, Where C_0 and C (mg/L) was the initial concentration and concentration at certain time intervals of solution, respectively.

Equ2 removal efficiency (%) = $(C_0-C)/C_0 \times 100\ \%$ (S2)

To discuss the effects of concentrations and adsorbent dosage on the adsorption properties, various BF solutions and different dosage of **1** were prepared with different concentrations of 5 mg/L, 10 mg/L, 15 mg/L and 0.2 mg/mL, 0.4 mg/mL, and 0.8 mg/mL, respectively.

To evaluate the removal efficiency of **1** for different dyes, both cationic dyes (MB, GV and

BF) and anionic dyes (MO) were used in the adsorption experiments. The dye concentrations were measured using an UV–vis spectrophotometer at 664, 584, 542, and 464 nm, respectively, in order to assess the removal efficiency of **1** for different dyes. Moreover, the pH values were adjusted using diluents such as HCl or NaOH.

The adsorption kinetic behavior of POM composite toward organic dyes were investigated by liner fitting using the pseudo-first-order, pseudo-second-order kinetic adsorption models and the Elovich equation in the following equation:

Pseudo-first-order kinetic model: $\ln(q_e\text{-}q_t)=\ln q_e\text{-}Kt$ (S3)

$q_e= \frac{C_0-C_e}{m} \times V$ (S4)

$q_t=\frac{C_0-C_t}{m} \times V$ (S5)

Pseudo-second-order kinetic model: $\frac{t}{q_t} =\frac{1}{K'qe^2}+\frac{t}{q_e}$ (S6)

The Elovich equation: $q_t= K_e \ln t + A$ (S7)

Where q_e and q_t (mg/g) are the amount of absorbed dyes at equilibrium and time t (min), V is the BF solution volume (mL); m is the mass of POM (g). K (1/min), K′ (g/(mg min)) and K_e are the pseudo-first-order, pseudo-second-order rate constants and the Elovich equation constant, the A is constant, respectively.

Four traditional isotherms, i.e., Langmuir, Freundlich, Dubinin-Radushkevich and the Temkin isotherm models are used to the adsorption isotherms of the as-prepared POM compound. The four models are described as:

Langmuir model: $\frac{C_e}{q_e} = \frac{1}{k_L q_{max}}+\frac{C_e}{q_{max}}$ (S8)

Freundlich model: $\ln q_e=\frac{1}{n} \ln C_e + \ln K_F$ (S9)

The Dubinin-Radushkevich model: $\ln q_e=\ln q_m\text{-}\beta\varepsilon^2$ (S10)

$\varepsilon=RT \ln(1+\frac{1}{C_e})$ (S11)

$E=\frac{1}{\sqrt{-2\beta}}$ (S12)

The Temkin isotherm model: $q_e=\frac{RT}{b} \ln K_t +\frac{RT}{b} \ln C_e$ (S13)

Where C_e (mg/L) is the equilibrium concentration, q_e (mg/g) is the adsorption capacity at equilibrium and q_{max} (mg/g) is the maximum amount under per unit weight of adsorbents, K_L (L/mg) is the Langmuir isotherm adsorption constant. K_F (mg/g(L/mg)$^{1/n}$) is the Freundlich isotherm adsorption constant, and 1/n corresponds to the adsorption intensity. where q_m (mg/g) is the amount of theoretical saturation adsorbed, β is the adsorption constant, ε is the Polanyi

potential and the value can be decided by the Eq. (S9), T (K) is the temperature in kelvin, R is the universal gas constant, E is the average free energy of adsorption and can be calculated by β. Kt is the Temkin isotherm constant, R (J/(mol*k)) is universal gas constant, T (K) is the thermodynamic temperature, b is constant relied on adsorption energy.

1.5 Antibacterial action investigation

1.5.1 SEM characterization of bacteria

A series of SEM images were performed to assess the influence of **1** on cell morphology of *E. coli* cells. Firstly, the treated and untreated bacteria cells were centrifuged, and then immobilized for 2 hours in 2.5% glutaraldehyde solution, followed by washing with PBS for 3 times. To dehydrate bacteria cells, each solution was retained for 15 minutes before being exposed to 50%, 70%, 80%, 90%, 95%, 100%, 100% ethanol solutions. Finally, bacteria cells samples were prepared with conductive tape and then observed with an SEM.

1.5.2 RNA leakage assay

It is generally possible to detect leakage of cellular content when the bacteria wall or membrane is injured and to estimate the amount of DNA and RNA released by assessing the absorbance of 260 nm [48]. The detailed experiments were as follows. Bacteria suspension were centrifuged for several minutes after incubation and soluted in 0.85% NaCl solution with the absorbance of 0.5 at 600 nm by adjusting the volume of bacteria. Then, the obtained bacterial suspension was incubated with **1** (4 mg) for 15 min at room temperature. Subsequently, the bacterial solution were filtered through 0.22 μM syringe filters to remove the bacteria and other impurities. Finally, UV-Vis spectrophotometer was employed to record the absorbance at 260 nm. Meanwhile, it can also be considered an indicator of bacterial cell membrane damage when the leakage of protein concentration is observed after the membrane of the bacterium is damaged, producing intracellular fractions such as protein, K^+, and others [49].

1.5.3 Protein leakage assay

The overnight grown culture of *E. coli* were suspended in 0.85% of saline solution (OD_{600} = 0.5). The cell suspensions were treated with 100 μg/mL concentration of composites for 1 h at 37 °C in a shaker. After incubation, the cells were collected by centrifugation and 1 mL of supernatant was withdrawn to quantify the relative protein leakage using Bradford protein assay method.

1.5.4 Assessment of reactive oxygen species (ROS)

The existence of oxidative stress as a significant antibacterial mechanism of antibacterial materials is also common, in addition to membrane stress caused by direct physical contact with the compound [46,50,51]. Therefore, reactive oxygen species and *in vitro* GSH oxidation were studied to examine the possibility of oxidative stress mediated by **1** [50]. By measuring the level of ROS in bacteria using the NBT reduction method [52] and it was possible to detect blue-violet formaldehyde produced at 575 nm in the bacterial cells. Firstly, 1 mL *E. coli* suspensions (10^8 CFU mL^{-1}) were cultured at 37 °C for 15 min with the treatment of **1** (4 mg) and the bacteria suspension without **1** was regarded as the control group. Afterwards, 4 mL NBT solution (1 $mg \cdot mL^{-1}$) was added into the above bacteria solutions and cultured at 37 °C for 30 min. Then, the reaction was suspended by droping 0.4 mL HCl (0.1 $mol \cdot L^{-1}$) and the suspensions were collected after abandoning the supernatants. Subsequently, 1 mL dimethyl sulfoxide (DMSO) was added into the bacteria suspensions in order to extract the reduced NBT. Ultimately, the bluish violet formazan was collected and the absorbance was detected at 575 nm.

1.5.5 Detection of thiols by Ellman's assay

The antioxidant property of glutathione allows it to act as an oxidative stress indicator for cells as it prevents oxidative stress damage to cellular components. Quantitative detection of the thiol groups in glutathione can be estimated at 412 nm absorbance by Ellman method [53]. Briefly, 4.0 mg **1** dissolved in bicarbonate buffer was dropped into 1 mL GSH (0.8 mM in bicarbonate buffer) to induce oxidation as the test group. GSH without the compound was considered as a negative control and GSH (0.8 mM) with the existence of H_2O_2 (1 mM) was regarded as a positive control. Each set of experiments was fully covered with tin foil and then incubation in a shaking flask for 15 min. Afterwards, Tris-HCl (0.05 M) and DTNB (Ellman's reagent, 5, 50-dithio-bis-(2-nitrobenzoic acid), Aladdin) (5:1) were added into the above solutions to obtain yellow production. Finally, the mixture was filtered by 0.22 μM syringe filters and the absorbance at 412 nm was recorded through spectrophotometer.

1.5.6 Evaluation of the respiratory chain dehydrogenases activity

The accumulation of ROS in bacteria not only leads to oxidative stress reactions, but also depresses enzyme activity [54]. In most case, Iodonitrotetrazole ammonium chloride (INT) can be

reduced to a dark red, fat-soluble compound by bacterial respiratory chain dehydrogenase (INF) in living cells. Thus, the content of INF at 490 nm can be determined by means of spectrophotometry to evaluate dehydrogenase activity within cells [46]. A detailed description of the experimental process can be found in the following. Generally, bacterial suspension (10^8 CFU mL^{-1}) with the presence of **1** was served as experimental group and control experiment was performed without the presence of **1**. The bacterial cells were completely inactivated by boiling for 20 min as the control group (–), while bacteria cells have native enzymatic activity and as the control group (+). 0.1 mL INT solutions (0.5%) were added into the above three suspensions and the mixtures were cultured under dark at 37°C for 15 min. Then, the reaction was suspended by adding in 50 μL formaldehyde and the bacteria were collected by centrifuging the suspensions. Subsequently, 250 μL solutions of acetone and ethanol (1:1) were employed to extract the INF for twice. Finally, the supernatants were obtained and the value of INF at 490 nm was detected by spectrophotometer.

2. Characterization

Fourier-transform infrared (FTIR) spectra in KBr pellets were performed using a using Bruker VERTEX 70 FT-IR spectrometer (Brooker Spectral Instruments, Germany) in the wavenumber range of 400–4000 cm^{-1} to investigate attachment of surface functional groups. X-ray diffraction (XRD) patterns were obtained using a Bruker D8 ADVANCE (Germany Bruker company, Germany) X-ray diffractometer (Cu Kα radiation, λ=1.5406 Å) in the range of 5–90 °C at a scan speed of 0.05 °/min to determine the crystal structure and phase purity. Ultraviolet-visible (UV-vis) absorption spectra were recorded using a TU-1900 spectrometer (Beijing Puanalysis General Instrument Co., Ltd, China) in the range of 190-800 nm with a resolution of 2 nm. Raman was performed using a RM5 spectrometer (Edinburgh Instruments Company, England) using the 532 nm laser line. X-ray photoelectron spectroscopy (XPS) measurements were performed using a Thermo ESCALAB 250 photoelectron spectrometer (American thermofisher scientific, Inc) to determine surface elemental compositions, chemical states and the binding energy charge was corrected to 284.6 eV for C1s. Field-emission scanning electron microscopy (FESEM) were acquired on JSM-7500 F SEM (Japan Electronics Corporation, Japan) with the equipment of energy-dispersive X-ray analysis (EDS) to study surface morphology and structure. Elemental analyses (C, H, and N) were performed on a PerkinElmer 2400 CHN elemental analyzer.

3. Crystal data of 1

Table S1. summary of crystal data and refinement results

**Crystal Data**	**1**
Empirical formula	$C_{28}H_{43}Ni_{2}Mo_{12}N_{20}O_{44}S_{4}P$
Formula weight	2791.66
Crystal system	Triclinic
Space group	*P-1*
T (K)	296(2)
a (Å)	11.1434(6)
b (Å)	12.6448(6)
c (Å)	13.1730(7)
α (deg)	104.549(2)
β (deg)	95.251(2)
γ (deg)	102.569(2)
V (Å^3)	1732.46(16)
Z	1
Dc (g·cm^{-3})	2.667
μ (mm^{-1})	2.877
2θ (deg)	4.436–50.198
F(000)	1333.0
Independent reflections	6106 [R_{int} = 0.0821, R_{sigma} = 0.0906]
Data/restraints/parameters	6106/48/522
R_1[a], wR_2[b] [$I \geq 2\sigma(I)$]	0.0696, 0.1620
R_1, wR_2 (all data)	0.0966, 0.1781
diff peak and hole, eÅ^{-3}	1.58/ −1.63

Table S2 The calculated results for Ni (II) ions configuration of **1** by SHAPE 2.1 software.

Configuration	ABOXIY, **1**
Hexagon (D_{6h})	33.392
Pentagonal pyramid (C_{5v})	23.055
**Octahedron (O_h)**	**2.593**
Trigonal prism (D_{3h})	11.854
Johnson pentagonal pyramid J2 (C_{5v})	27.722

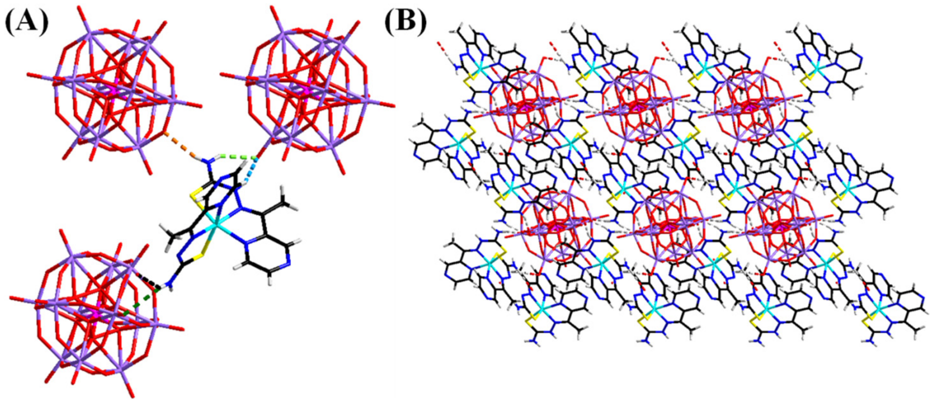

Figure S1. (A) Connect of hydrogen bond between $[Ni(L^2)(HL^2)]^+$ and $[PMo_{12}O_{40}]^{3-}$. (B) 2D layered structure of compound. color scheme: Mo: purple. P: pink. O: red. Ni: turquoise. S: yellow. N: blue. C: gray-50%

4. Stability measurement of 1

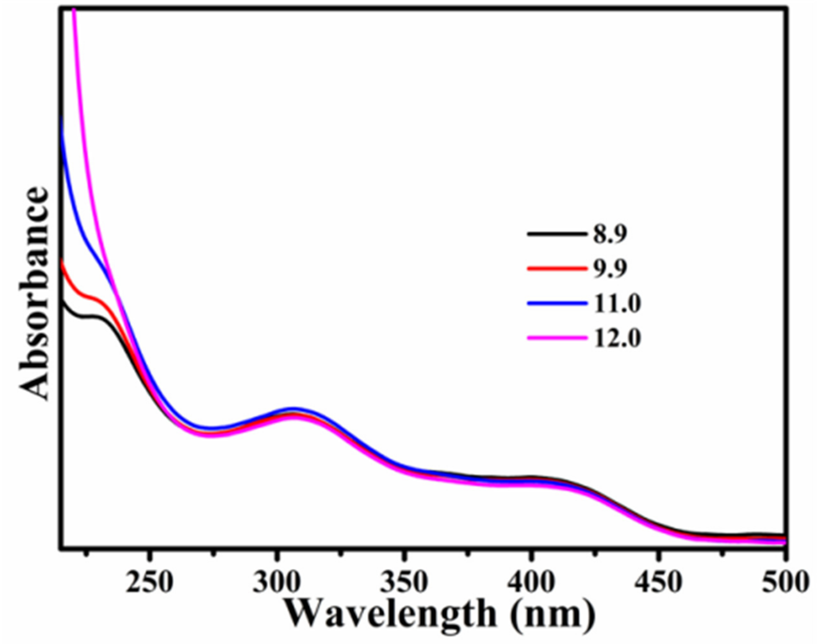

Figure S2. Stability measurement of **1**

5. Time-kill graph statistics of 1

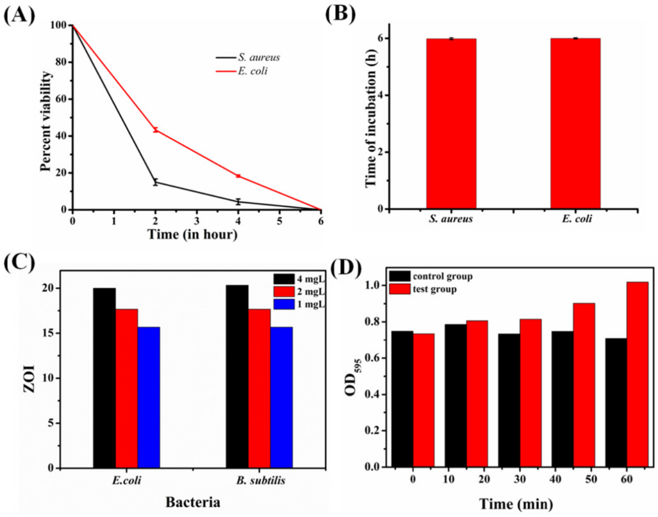

Figure S3. (A, B) Time kill graph statistics of **1.** (C) inhibition zone for *E coli* and *B.subtilis*. (D) evaluation of the protein leakage volume at 595 nm

6. Adsorption performance

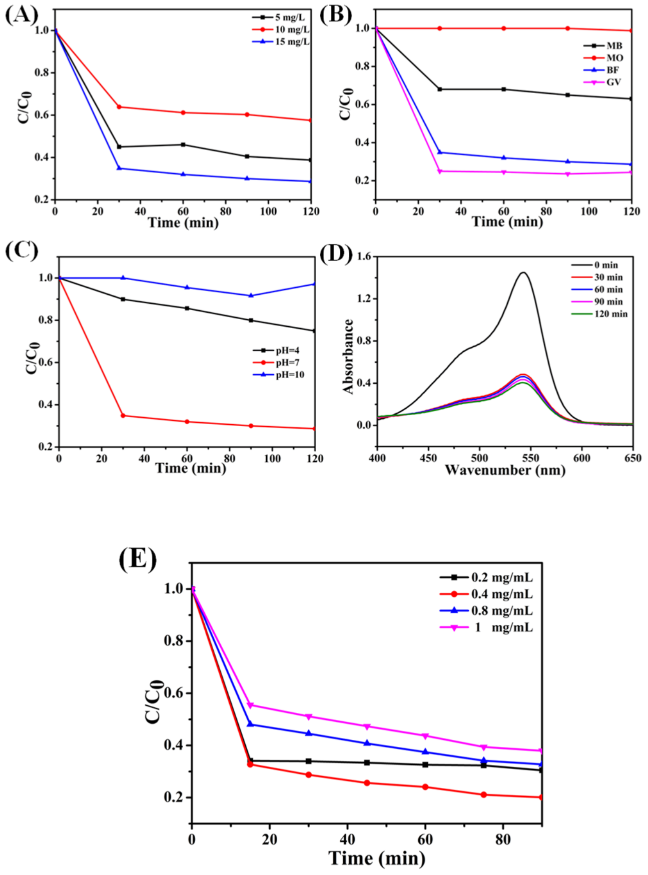

Figure S4. (A) adsorption performance of **1** at different concentration of BF dye for 120 min. (B) adsorption performance of various dyes. (C) adsorption performance of three different pH environments. (D) UV–vis spectra for BF dye solution. (E) Adsorption performance of **1** for BF solution with different adsorption concentration dosage (0.2 mg/mL, 0.4 mg/mL, 0.8 mg/mL, and 1 mg/mL)

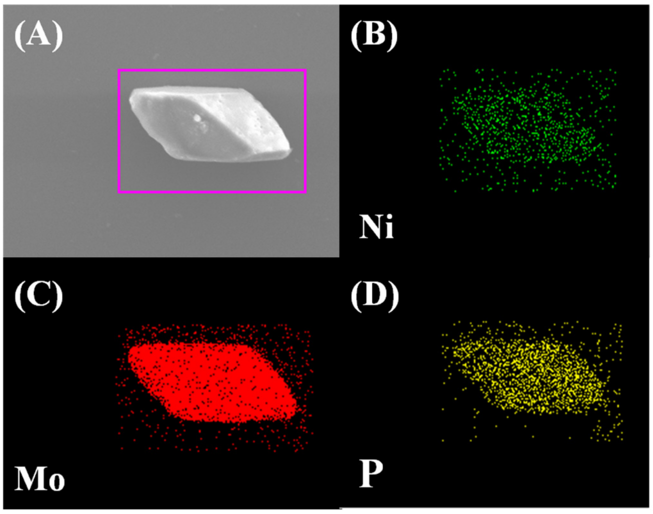

Figure S5. (A) The SEM image of **1** after adsorption; (B-D) the SEM elemental mapping of Ni, Mo, and P in **1** after adsorption

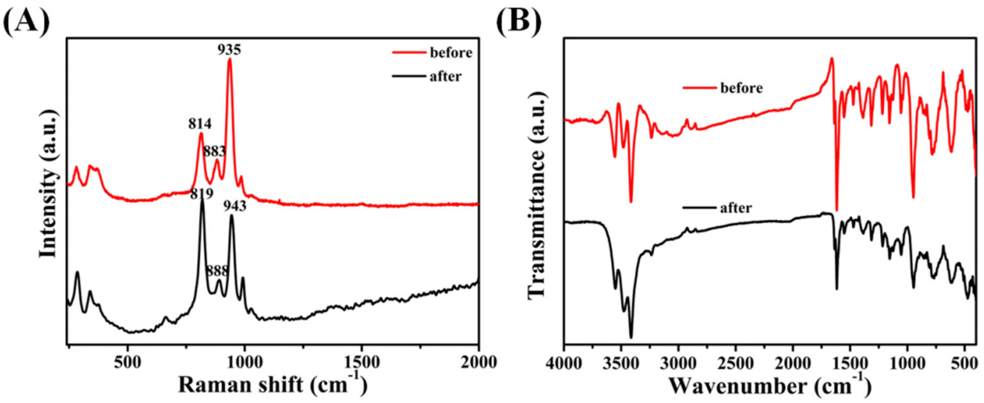

Figure S6. (A) Raman spectrum of **1** before and after adsorption; (B) IR spectrum of **1** before and after adsorption

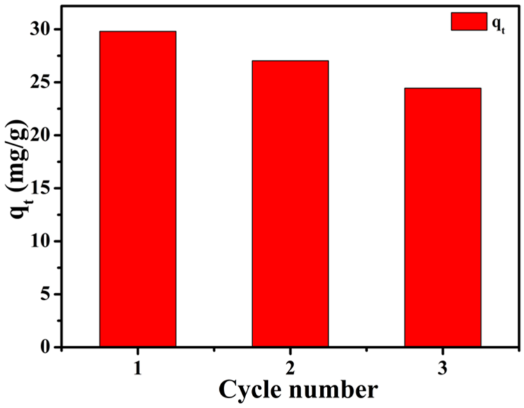

Figure S7. Effect of regeneration cycles on the adsorption capacity of BF dyes onto **1**
